# Plague vaccine: recent progress and prospects

**DOI:** 10.1038/s41541-019-0105-9

**Published:** 2019-02-18

**Authors:** Wei Sun, Amit K. Singh

**Affiliations:** Department of Immunology and Microbial Disease, Albany Medical College, Albany, NY 12208 USA

## Abstract

Three great plague pandemics, resulting in nearly 200 million deaths in human history and usage as a biowarfare agent, have made *Yersinia pestis* as one of the most virulent human pathogens. In late 2017, a large plague outbreak raged in Madagascar attracted extensive attention and caused regional panics. The evolution of local outbreaks into a pandemic is a concern of the Centers for Disease Control and Prevention (CDC) in plague endemic regions. Until now, no licensed plague vaccine is available. Prophylactic vaccination counteracting this disease is certainly a primary choice for its long-term prevention. In this review, we summarize the latest advances in research and development of plague vaccines.

## Introduction

Plague is caused by the facultative, intracellular Gram-negative bacterial pathogen, *Yersinia pestis*. As one of the oldest and most notorious infectious diseases, plague’s notoriety came from the estimated 200 million deaths that were claimed throughout recorded human history, and the extensive devastation that was imparted on societies which subsequently shaped the progress of human civilization.^1,2^ Currently, plague is less active than other well-known infectious diseases, e.g., AIDS, malaria, influenza, tuberculosis, dengue, and certain antibiotic-resistant superbugs (http://www.who.int/news-room/fact-sheets). However, its role as a serious public health concern should not be relegated to antiquity. Lingering fears of future outbreaks are justifiable as plague persists in rodent hosts, has significantly increased its geographical range, remains endemic to many regions around the globe, and is responsible for several thousand annual human cases worldwide.^3^ In 2015, 15 human cases of plague were reported in the US, resulting in 4 deaths^4^ and in late 2017, the island of Madagascar had experienced a large outbreak of plague, where a total of 2348 confirmed, probable and suspected cases of plague (~70% are pneumonic form) occurred, including 202 deaths (case fatality rate 8.6%),^5–7^ inciting regional panics. Moreover, there are increasing concerns of multiply antibiotic resistant *Y. pestis*^8–12^ due to the intrinsic genetic plasticity of bacterium.^13,14^ Thus, plague is internationally recognized as a re-emerging disease.^15–17^

Additionally, *Y. pestis* has been used intentionally as a biological weapon clearly recorded in human history,^5,6^ and is considered one of the most likely biothreat agents.^7,8^ During the Cold War, the Centers for Disease Control and Prevention (CDC) recognized aerosolized *Y. pestis* as a potent biological weapon, and classified the bacteria as a tier 1 select agent.^18^ In nature, following the bite of an infected flea, the mammalian host will typically manifest infection in the bubonic form, and may develop septicemic or secondary pneumonic infection if not promptly treated. Direct inhalation of aerosolized *Y. pestis* can result in an extremely lethal form of primary pneumonic plague.^1^ The short incubation period (1–3 days) of pneumonic plague allows rapid disease progression with a high fatality rate, and historically, victims often become sources of secondary infections as the disease spreads throughout a population.^1,4^

As a countermeasure against the above scenarios, it is imperative to develop a safe and efficacious vaccine against plague. Vaccination is believed to be an efficient strategy for long-term protection. Previous reviews have comprehensively summarized different kinds of plague vaccine developments, including live recombinant, subunit, vectored, and other formulated vaccines before 2016 (see reviews^19–32^). Here, we only update the most recent advances of vaccine development (listed in Table 1) and assess the likely prophylactic and therapeutic plague vaccines.

**Table 1:** Vaccine evaluation against plague

Vaccine candidates	LD_50_	Immunization	Protective efficacy	Reference
F1-LcrV-HSP70(II) fusion protein	ND	Female BABL/C mice vaccinated s.c. with 20 μg/mouse	Complete protection against i.p. challenge with 100 LD_50_ (10^5^ CFU) of *Y. pestis* S1 strain	^44^
rF-V1 adjuvanted with a novel TLR4 ligand, BECC438	ND	Female C57BL/6J mice vaccinated s.c. with 20 μg/mouse	complete protection against i.p. challenge with ∼20 × LD_50_ of *Y. pestis* CO92 Δ*pgm*	^47^
Flagellin/F1/V	ND	healthy individuals aged 8 through 45 years by i.m. injection	ND	^48^
F1mutV-PA	ND	Female Balb/c mice and Brown Norway rats immunized by the i.m. route with 50 µg of F1mutV-PA and were boosted once on day 21	Complete protection of mice against *simultaneous* challenge with 200 LD_50_ *Y. pestis* CO92 (i.n.) and 1 LD_100_ lethal toxin of *B. anthracis (i.v.) and c*omplete protection of rats against *simultaneous* challenge with 400 LD_50_ *Y. pestis* CO92 (i.n.) and 1 LD_100_ lethal toxin of *B. anthracis (i.v.)*	^49^
VypVaxDuo	ND	BALB/c mice immunized in the dual route dosing regimen on d. 0, 21 with F1/Gln + V/His PCMC s.c. and boosted orally with formulation B	full protection for BALB/c mice against the s.c. challenge with 2 × 10^4^ LD_50_ of *Y. pestis* CO92	^50^
F + rV (composed of native F1, extracted from *Y. pestis*, and the recombinant V antigen)	ND	Cynomolgus macaques and Human adults	Induced a robust immune response up to 12 months and showed a good safety profile in both Cynomolgus macaques and Human adults	^51, 52^
Δ*nlpD Y. pestis* Kimberley53	>10^7^ CFU for s.c and airway routes of infection in Female OF1 mice	s.c. immunization with 10^7^ CFU of mutant strain	Provides complete protection against s.c. challenge with 10^5^ LD_50_ of *Y. pestis* Kimberley53 and 82% protection against i.n. challenge with 5500 CFU of *Y. pestis* Kimberley53	^53^
Δ*nlpD Y. pestis* 231 Δ*nlpD Y. microtus* I-3455 and Δ*nlpD Y. microtus* I-2359	All were avirulent in mice upon s.c. administration to BALB/c mice (100% survived the infection at a dose of 102, 103, 105, and 107 CFU), and in guinea pigs (100% survival rate at a dose of 1.5 × 10^10^ CFU)	s.c. immunization with each mutant strain	Immunization with the Δ*nlpD* mutant was generated in several *Y. pestis* strains (subsp. *Y. pestis* bv. antiqua, subsp. microtus bv. aitaica) and provided potent immunity against plague in the mouse model), but failed to do so in the guinea pig model	^54^
*Y. pestis* CO92 Δ*rbsA* Δ*lsrA*	80–100% of female Swiss Webster mice surviving a challenge dose of 8- to 50-LD_50_ equivalent of WT CO92	ND	ND	^55^
*Y. pestis* CO92 Δ*lpp* Δ*msbB* Δ*ail* or Δ*lpp* Δ*msbB::ailL2*	>2.5 × 10^6^ CFU by s.c. infection and >5 × 10^6^ CFU CFU by i.n. infection female Swiss Webster mice	i.m. route with two doses (2 × 10^6^ CFU/dose) *Y. pestis* CO92 Δ*lpp* Δ*msbB* Δ*ail* or Δ*lpp* Δ*msbB::ailL2* at 0 and 21 days	On day 120, mice were challenged via the i.n. route with 1.2 × 10^4^ CFU dose (24 LD_50_) of the WT CO92 *luc2* strain, 80% animal survival	^56^
*Y. pestis* CO92 Δ*lpp* Δ*msbB* Δ*ail*	>2.5 × 10^6^ CFU by s.c. infection Brown Norway rats	i.m. route with two doses (2 × 10^6^ CFU/dose) *Y. pestis* CO92 Δ*lpp* Δ*msbB* Δ*ail* at 0 and 21 days	i.n. route challenge with WT CO92 *luc2* strain at the dose of either 2.3 × 10^4^ CFU (46 LD_50_) on day 43 to evaluate short-term protection or 1.6 × 10^4^ CFU (31 LD_50_) on day 91 to evaluate long-term protection. 100% survival for immunized rats	^56^
*Y. pestis* CO92 Δ*lpp* Δ*msbB* Δ*pla*	>2.5 × 10^6^ CFU by s.c. infection and > 5 × 10^6^ CFU CFU by i.n. infection female Swiss Webster mice	i.m. route with two doses (2 × 10^6^ CFU/dose) *Y. pestis* CO92 Δ*lpp* Δ*msbB* Δ*pla* at 0 and 21 days	On day 120, mice were challenged via the i.n. route with 1.2 × 10^4^ CFU dose (24 LD_50_) of the WT CO92 *luc2* strain, all animal survival	^56^
*Y. pestis* CO92 Δ*lpp* Δ*msbB* Δ*pla*	>2.5 × 10^6^ CFU by s.c. infection Brown Norway rats	i.m. route with two doses (2 × 10^6^ CFU/dose) *Y. pestis* CO92 Δ*lpp* Δ*msbB* Δ*pla* at 0 and 21 days	i.n. route challenge with WT CO92 *luc2* strain at the dose of either 2.3 × 10^4^ CFU (46 LD_50_) on day 43 to evaluate short-term protection or 1.6 × 10^4^ CFU (31 LD_50_) on day 91 to evaluate long-term protection. 100% survival for immunized rats	^56^
*Y. pestis* CO92 Δ*lpp*Δ*cyoABCDE*,	90% survival of female Swiss Webster mice by i.n. infection with 11 LD_50_ of *Y. pestis* CO92	Survival mice re-challenge	50% survival of female Swiss Webster mice by i.n. infection with 10 LD_50_ of *Y. pestis* CO92	^57^
*Y. pestis* CO92 Δ*vasK*Δ*hcp6*	60% survival of female Swiss Webster mice by i.n. infection with 9 LD_50_ of *Y. pestis* CO92	Survival mice re-challenge	40% survival of female Swiss Webster mice by i.n. infection with 8 LD_50_ of *Y. pestis* CO92	^57^
*Y. pestis* CO92 Δ*ypo2720-2733*Δ*hcp3*	60% survival of female Swiss Webster mice by i.n. infection with 9 LD_50_ of *Y. pestis* CO92	Survival mice re-challenge	60% survival of female Swiss Webster mice by i.n. infection with 8 LD_50_ of *Y. pestis* CO92	^57^
*Y. pestis* EV76 and virulent *Y. pestis* KIM53 co-infection	ND	C57BL/6 mice	Simultaneous co-administration of the EV76 and virulent KIM53 provided 91% protection for mice by s.c. challenge with 100 CFU of KIM53 stain and injection with EV76 at 5 h post-challenge with 100 CFU of KIM53 stain could rescue survival of 34% mice	^58^
VTnF1	LD_50_ of the VTnF1 strain in OF1 female mice is more than 10^9^ CFU	Oral immunization with 10^8^ CFU of VTnF1 strain	Conferred 100% protection against pneumonic plague using a high-dose challenge (3300 LD_50_) caused by the fully virulent *Y. pestis* CO92. Moreover, vaccination protected 100% of mice from bubonic plague caused by a challenge with 100 LD_50_ *Y. pestis* and 93% against a high-dose infection (10,000 LD_50_)	^66, 126^
χ10069(pYA5199) (Δ*asd-206* Δ*yopJ315*Δ*yopK108)* harboring an Asd + plasmid to deliver LcrV via Type three secretion system (YopE_Nt138_-LcrV^67^)	LD_50_ of the χ10069(pYA5199) strain in Swiss Webster mice is more than 10^9^ CFU	Single dose oral immunization with 10^9^ CFU of χ10069(pYA5199) strain	Provide 90% protection against i.n. challenge with 5 × 10^4^ CFU of virulent *Y. pestis KIM6+* *(pCD1Ap)* strain at 35 days post immunization	^Manuscript in preparation^
Live attenuated *S*. Typhimurium mutant strain, χ12094(pYA5383) delivering three protective antigens (LcrV, F1 and Psn)	10^9^ CFU of χ12094(pYA5383) did not caused any deaths or other disease symptoms in SCID mice over a 60-day period	Oral immunization with 10^9^ CFU of χ12094(pYA5383) and oral booster with same dose of χ12094(pYA5383)	complete protection against s.c. challenge with 5700 CFU (~570 LD_50_) of *Y. pestis* CO92 and 60% protection against intranasal challenge with 5000 CFU (~50 LD_50_) of *Y. pestis* CO92	^68^
*F. tularensis* LVS Δ*capB*/Yp	ND	Homologous priming-boosting with LVS Δ*capB*/Yp by intradermal (i.d.) route	50% protection against intranasal challenge with 1900 CFU *of Y. pestis* CO92 (~8 LD_50_)	^69^
*F. tularensis* LVS Δ*capB*/Yp plus *L. monocytogenes* Δ*actA* Δ*inlB prfA*/Yp	ND	heterologous priming-boosting with rLVS Δ*capB*/Yp by i.n. route and rLm Δ*actA* Δ*inlB prfA*/Yp by intramuscular (i.m.) route	50% protection against intranasal challenge with 1900 CFU *of Y. pestis* CO92 (~8 LD_50_)	^69^
A replication-defective human type 5 adenovirus (Ad5) vector to express the codon-optimized fusion gene *YFV* (*ycsF*, *caf1*, and *lcrV*)	ND	Female Swiss-Webster mice and nonhuman primates Cynomolgus macaques immunized with Ad5-Empty by i.m. at day 0, rAd5-YFV by i.n. at day 30 and boosted with 50 μg of rYFV at day 42	Complete protection for mice against aerosolized *Y. pestis* CO92 at a Dp of 4.62 × 10^5^ CFU and Complete protection for NHPs against the aerosolized WT CO92 at Dp ranging from 1.32 × 10^7^ to 8.08 × 10^7^ CFU	^70^
*L. plantarum* delivering LcrV fused with the lipidation motif of OspA protein of *B. burgdorferi*	ND	Oral vaccination with lipLcrV-*L. plantarum* followed by two boosts	No any protection against i.n. challenge with 10 or 100 LD_50_ of *Y. pestis* CO92 pgm^−^	^71^
TMV delivering LcrV and F1		I.N. vaccination and boost with TMV-LcrV + TMV-F1	complete protection against morbidity and mortality associated with pneumonic infection with 10 × LD_50_ *Y. pestis* CO92pgm^−^	^71^
Sylvatic plague vaccine [RCN-F1/V307])	ND	Field trials	Partially Protects for Prairie Dogs (*Cynomys* spp.) in Field Trials	^75^
Monoclonal antibody F2H5	ND	BALB/c mice received 100 μg of monoclonal antibody via tail vein injection 24 h before the *Y*. *pestis* challenge	complete protection against subcutaneous *Y*. *pestis* infection	

*Y. pestis* KIM6 + (pCD1Ap), LD_50_ (s.c.) < 10 CFU, LD_50_ (i.n.) ~ 100CFU^127^; *Y. pestis* Kimberley53, LD_50_ (s.c.) 1–3 CFU, LD_50_ (i.n.) = 550 CFU^53^; *Y. pestis* CO92, LD_50_ (s.c.) = 1.9 CFU, LD_50_ (i.n.) ~ 2_50_ CFU, LD_50_ (aerosol) ~ 2100 CFU^97,128,129^ *Y. pestis* CO92Δ*pgm* strain, LD_50_ (i.n.) = 2 × 10^4^ CFU^130^

*CFU* colony forming units, *LD*_*50*_ 50% lethal dose, *s.c.* subcutaneous, *i.n.* intranasal, *i.p.* intraperitoneal, *i.m.* intramuscular, *ND* not detected

## Subunit vaccine

Many studies have established that the low calcium response protein V (LcrV), a multifunctional virulence protein, is an indispensable protective antigen against *Y. pestis* infection.^24,28,33^ Vaccine research found that recombinant LcrV, alone or combination with F1, in mixed cocktail and fusion formats, was able to provide superior protection against bubonic and pneumonic plague infections in different animal models (i.e., mice, rat, guinea pig, and Cynomolgus macaques).^34–37^ Clinical trials of LcrV and F1 subunit vaccines (RypVax™ and rF1^−^V) began around a decade ago.^27^ RypVax™ manufactured by PharmAthene Inc. was a recombinant plague vaccine comprising separate recombinant F1 (rF1) and V (rV) antigens produced in *Escherichia coli* (http://media.corporate-ir.net/media_files/irol/19/191999/FactSheet-RypVax-Oct2008.pdf). The rF1-V fusion vaccine was developed by The United States Army Medical Research Institute for Infectious Diseases (USAMRIID)^38^ and currently being further developed by Dynport Vaccine Company, LLC.^27^ The rV10, a truncated LcrV antigen developed by Schneewind’s group in 2011, is currently undergoing US Food and Drug Administration (FDA) pre-Investigational New Drug authorization review for a future phase I trial.^27^ In comparison to rF1-V, immunization with rV10 revealed no substantial differences in protection efficacy against pneumonic plague infection in mice, guinea pigs or Cynomolgus macaques. However, both rF1-V^39,40^ and rV10^34^ vaccines were unable to protect African green monkeys against pneumonic plague uniformly as Cynomolgus macaques, despite eliciting robust antibody response. The inconsistent efficacy of these subunit vaccines in African green monkeys and Cynomolgus macaques was speculated to be due to a deficiency in innate or cellular immunity, resulting in a lack of effective synergistic action between humoral and cell-mediated immune response to defend against pneumonic plague.^41^ Recently, several groups are trying to enhance immunogenicity of the subunit vaccines using different means.

The heat shock protein 70 domain II [HSP70(II)] of *Mycobacterium tuberculosis* as an immunomodulator was able to stimulate effective T-cell responses^42^ and ovalbumin-HSP70(II) fusion protein was sufficient to elicit ovalbumin specific CD8+ cytotoxic T lymphocytes.^43^ Based on these findings, Tuteja’s group^44,45^ fused the F1 and LcrV antigens of *Y. pestis* with the HSP70(II) [F1-LcrV-HSP70(II) protein] as a plague vaccine to enhance cell-mediated immune response. A group of BALB/c mice immunized with F1-LcrV-HSP70(II) protein had significantly increased percentages of CD4+ and CD8+ T cells producing IL-2, TNF-α, and IFN-γ in comparison to the group of mice immunized with F1-LcrV fusion protein. However, immunization either with F1-LcrV-HSP(II) or F1-LcrV afforded complete protection for mice against intraperitoneal (i.p.) challenge with 100 LD_50_ of virulent *Y. pestis* S1 strain. A possible reason is that the lower dose of i.p. challenge might not differentiate the protective efficacy contributed from cellular immunity elicited by F1-LcrV-HSP(II).

Gregg et al.^46^ generated an *Y. pestis* KIM6+ derived mutant strain, *Yp* Δ*msbB pagPYp*^Rep^, in which the mutant disrupts the secondary lauryl acyl-transferase (MsbB) and restores the palmitate transferase (PagP) of *Y. pestis*. The mutant strain yielded a structurally distinct lipooligosaccharide molecule (BECC438) that can elicit Toll-like receptor 4 (TLR4) activation. C57BL/6J mice intramuscularly (i.m.) immunized with BECC438 adjuvated rF1-V using a prime-boost regimen were fully protected against i.p. challenge with ∼20 × LD_50_ of *Y. pestis* CO92 Δ*pgm* strain.^47^

Intramuscular injection of Flagellin/F1/V in a dose escalation manner was conducted in healthy individuals from aged 8 through 45 years in a phase I trial. Sixty healthy subjects were enrolled; 52% males, 100% non-Hispanic, 91.7% white and mean age 30.8 years. Positive antibody responses were observed to F1, V, and flagellin with no severe reactogenicity.^48^ Rao’s group has developed a rF1mutV-PA recombinant subunit vaccine consisting of *Y. pestis* F1 and LcrV dual antigens, and *Bacillus anthracis* protective antigen (PA) adjuvanted with Alhydrogel®.^49^ The tri-valent vaccine elicited robust antibody responses in mice, rats, and rabbits and conferred complete protection in mice and rats against simultaneous intranasal (i.n.) challenge with *Y. pestis* CO92 and lethal intravenous (i.v.) injection of *B. anthracis* toxin.^49^ The F1mutV-PA was the first subunit vaccine showing complete protection against simultaneous challenge with *Y. pestis* and lethal *B. anthracis* toxin challenge in a variety of animal models, and demonstrated a potential prophylactic vaccine for preventing a bioterror attack with weaponized *B. anthracis* and/or *Y. pestis*.^49^

VypVaxDuo is a novel vaccine developed by Moore et al.^50^ and composed of the recombinant F1 and V proteins mixed with different formulations using a subcutaneous (s.c.) prime and an oral booster regimen. An early onset antibody response (IgG and IgA) was observed 14 days post-primary immunization, and full protection against s.c. challenge with 2 × 10^4^ LD_50_ of *Y. pestis* CO92 was observed upon regimen completion in BALB/c mice. Moreover, Moore et al. approached their vaccine design with the goal of creating a practical solution for low- and middle-income countries endemic to plague. In this regard, VypVaxDuo is a strong potential vaccine as the primary vaccine formulation was exceptionally stable in vialled form under thermostressed conditions, circumventing the need for a cold chain for distribution and storage. Additionally, the prime-boost regimen requires only one clinic visit for the s.c. priming vaccination, as the oral boost vaccine formulation can be self-administered and minimizes the need for medical personnel and intervention.

A novel subunit plague vaccine developed by Liu et al. is composed of a native F1 and recombinant V (F1 + rV) antigens absorbed to aluminum hydroxide adjuvant. The F1 + rV vaccine induced a very strong humoral immune response and a low level of cell-mediated immune response in cynomolgus macaques.^51^ Subsequently, the National Institutes for Food and Drug Control (NIFDC) and the Jiangsu Provincial Centers for Disease Control and Prevention (CDC) conducted a one-year immunogenicity and vaccine safety study where 240 healthy adults aged 18–55 years were F1 + rV-immunized with 15 μg at day 0 or 20 μg at day 28. Results showed that anti-F1 titers and seroconversion rates were maintained at high levels up to 12 months, while anti-V titers and seroconversion rates decreased sharply at 6 months and continued to decrease at 12 months. No vaccine-related serious adverse events were observed during immunization. Overall, human clinical trials show the F1 + rV subunit vaccine induces a robust humoral immune response up to 12 months and has a good safety profile in humans.^52^

## Attenuated *Yersinia* vaccine

Lipoprotein NlpD of *Y. pestis* is an essential virulence factor for the development of bubonic and pneumonic plague.^53,54^ Subcutaneous administration of the Δ*nlpD Y. pestis* Kimberley53 mutant conferred protection to mice against bubonic and pneumonic plague better than the EV76 vaccine strain.^53^ Dentovskaya et al. generated a variety of Δ*nlpD* mutant strains based on three *Yersinia* parental strains (i.e., subsp. *pestis* bv. antiqua strain 231; subsp. *microtus* bv. altaica strains I-3455 and I-2359). In comparison to the reference vaccine strain EV NIIEG, immunization with the Δ*nlpD* mutant strains provided potent protective immunity against plague in BALB/c mice challenged with 200 LD_100_ of virulent *Y. pestis* 231 strain, but failed to do so in the guinea pig model.^54^ The intrinsic reasons are not clear yet, but the inconsistent protection observed in different animal models diminishes the possibility of Δ*nlpD Y. pestis* mutant as one of the live plague vaccine candidates.

Chopra’s group characterized effects of the conserved quorum-sensing system (autoinducer-2, AI-2) on pulmonary *Y. pestis* infection in mice.^55^ In a series of mouse studies, they demonstrated that the deletion of ABC transport systems components (*rbsA* and *lsrA* genes) synergistically disrupted AI-2 signaling patterns and reduced more than 50-fold virulence of *Y. pestis* strain CO92 by pulmonary challenge in mice. However, deletion of *luxS* or *lsrK* (encoding AI-2 kinase) on top of the Δ*rbsA* Δ*lsrA* background strain restored the virulence phenotype as that of the wild-type *Y. pestis* CO92 or the Δ*rbsA* Δ*lsrA* mutant complemented with the *rbsA* and *lsrA* genes. The administration of synthetic AI-2 in mice could rescue the virulence of Δ*rbsA* Δ*lsrA* Δ*luxS* mutant equal to that of the Δ*rbsA* Δ*lsrA* strain, but couldn’t rescue the virulence of AI-of Δ*rbsA* Δ*lsrA* Δ*luxS* Δ*lsrK* mutant.^55^ More recently, the same group evaluated the long-term immunity of the *Y. pestis* mutant strains Δ*lpp*Δ*msbB*Δ*ail* and Δ*lpp*Δ*msbB::ailL2* (Δ*lpp* lacks the Braun lipoprotein, Lpp; Δ*msbB* lacks an acetyltransferase, MsbB; *Δail* lacks the attachment invasion locus, Ail; *ailL2* is a modified Ail with diminished virulence). Immunization of mice and rats with *Y. pestis* Δ*lpp* Δ*msbB* Δ*ail*, Δ*lpp* Δ*msbB::ailL2* or Δ*lpp* Δ*msbB* Δ*pla* mutations generated long-term humoral and cellular immune responses and afforded comprehensive protection against pulmonary challenge of *Y. pestis* CO92 on day 120.^56^ Due to high attenuation of *Y. pestis* Δ*lpp* Δ*msbB* Δ*pla* mutant in mice and rats, the strain was recently excluded from the Centers for Disease Control and Prevention select agent list.^56^ In a subsequent study, Chopra’s group tested additional mutants with combinations of different gene deletions based on results from an in vivo signature-tagged mutagenesis (STM) screening, and found that immunization with these mutant strains conferred protection against pneumonic plague of varying levels.^57^

Zauberman et al. assessed whether immunization with the EV76 live vaccine can stimulate rapid and effective protective immunity against immediate challenge of virulent *Y. pestis* KIM53 strain. C57BL/6 mice were s.c. challenged with 100 CFU (100 LD_50_) of virulent KIM53; s.c. immunization with 10^7^ CFU of EV76 at the time of challenge conferred 91% protection, whereas s.c. immunization at 5 h post-challenge conferred 34% protection. Subsequently, the group assessed whether EV76-administration might promote rapid protection against pneumonic plague. C57BL/6 mice were s.c. immunized with 1 × 10^7^ CFU of EV76, then i.n. challenged with 1 × 10^4^ CFU (10 LD_50_) of KIM53 either concomitantly or 2 days post-immunization (dpi). The concomitantly immunized mice merely extended survival duration from 3 to 6.8 days, ultimately succumbing to infection, whereas the 2 dpi challenged mice had a 60% survival rate. *Ex vivo* analysis of *Y. pestis* growth in serum derived from EV76-immunized mice revealed that the rapid antibacterial activity was mediated by host heme- and iron-binding proteins hemopexin and transferrin, resulting in iron deprivation and further limiting the propagation of virulent *Y. pestis* in the host milieu, a form of host defense termed nutritional immunity.^58^ Based on current studies,^59–62^ vaccination with EV76 strain elicits a rapid and potent innate immune memory that could potentially provide considerable and immediate protection against bubonic and pneumonic plague, prior to mounting an adaptive immune response, which supports a novel therapeutic strategy for post-outbreak emergency responses.

The less virulent ancestor to *Y. pestis*,^63^ *Y. pseudotuberculosis*, typically causes a limited enteric disease in human and animals. *Y. pestis* and *Y. pseudotuberculosis* are remarkably similar in that they are >95% genetically identical and share a virulence plasmid, and they are different in that *Y. pestis* carries the additional plasmids pPCP1 and pMT1.^64^ Therefore, recombinant attenuated *Y. pseudotuberculosis* strains as a plague vaccine would be safer alternatives. Demeure’s group and our group developed different attenuated *Y*. *pseudotuberculosis* either heterologous synthesizing capsule antigen F1^65,66^ or delivering LcrV by Type three secretion system.^67^ Both groups demonstrated that a single dose of oral immunization with live attenuated *Y*. *pseudotuberculosis* induced potent antibody and cell-mediated responses, and significant Th17 response in mice, and moreover provided significant protection against pulmonary challenge with high-dose virulent *Y. pestis* strains.^65–67^ However, protective efficacy and safety of these live attenuated *Y*. *pseudotuberculosis* strain should be evaluated further in other animal models. Altogether, those recent studies contribute to the growing evidences supporting development of live *Yersinia* vaccines as countermeasures for preventing plague.

## Live vectored plague vaccines

An improved Recombinant Attenuated *Salmonella* Typhimurium Vaccine (RASV) strain expressing multiple plasmid-encoded *Y. pestis* antigens, including LcrV196 (aa residues 131–326), Psn (pestisin receptor) and F1, has been studied by our group. Synthesis of multiple antigens did not cause adverse effects on bacterial growth. BALB/c mice were orally immunized with the RASV strain, χ12094(pYA5383). High antibody titers specific for rLcrV, Psn, and F1 were developed. Complete protection was conferred against s.c. challenge with 5700 CFU (~570 LD_50_) of *Y. pestis* CO92, and 60% survival against i.n. challenge with 5000 CFU (~50 LD_50_) of *Y. pestis* CO92.^68^ Oral immunization with χ12094(pYA5383) did not caused any deaths or disease symptoms in SCID mice over a 60-day period.^68^

Horwitz’s group investigated an *F. tularensis* LVS Δ*capB* mutant strain and an attenuated *Listeria monocytogenes* (Lm) strain as vectors to deliver multiple protective antigens from *B. anthracis* and *Y. pestis* as a novel vaccine platform to combat three Tier 1 select agents, *B. anthracis*, *Y. pestis*, and *F. tularensis*.^69^ Homologous prime-boost with the LVS Δ*capB*-vectored vaccines or heterologous prime-boost with LVS Δ*capB* and Lm-vectored vaccines induced robust antigen-specific humoral immune responses, conferred protective immunity against lethal pulmonary challenge with *B. anthracis* Ames spores and *F. tularensis* Schu S4, but only afforded 50% protection against intranasal challenge with 1900 CFU *of Y. pestis* CO92 (~8 LD_50_).^69^ This study provided a proof of concept for an all-in-one vaccine providing protection against several tier 1 pathogens simultaneously.

In addition, Chopra’s group utilized a replication-defective human type 5 adenovirus (Ad5) vector for expression of a codon-optimized fusion gene *YFV* (*ycsF*, *caf1*, and *lcrV*). A heterologous prime-boost of mice and cynomolgus macaques with the trivalent rAd5-YFV vaccine conferred 100% protection against a stringent aerosol challenge dose of *Y. pestis* CO92.^70^ Arnaboldi et al. evaluated two distinct mucosal delivery platforms, a live bacterial vector, *Lactobacillus plantarum*, and a tobacco mosaic virus (TMV) vector for the intranasal administration of LcrV and F1 antigens.^71^ Both LcrV/F1-expressing vectors induced similarly high titers of IgG antibodies and proinflammatory cytokine secretion. Only the TMV-conjugated LcrV or F1, however, protected against subsequent lethal challenge with *Y. pestis*. These results suggest that mucosal delivery of TMV synthesizing F1-LcrV might induce complete protection against a lethal pneumonic infection of *Y. pestis* in mice.

Researchers at the United States Geological Survey’s National Wildlife Health Center have developed a Sylvatic Plague Vaccine (SPV) comprised of raccoon poxvirus (RCN) expressing both F1 and truncated V protein (V307) antigens, designed as a bait vaccine to protect Prairie dogs (*Cynomys spp*.).^72,73^ Prairie dogs are highly susceptible to *Y. pestis* and as such are potential sources of plague transmission to humans.^74^ Most recently, field trials showed that consumption of SPV-laden baits can protect prairie dogs against plague,^75,76^ which offers an additional approach for controlling plague transmission in epidemic areas.

Outer membrane vesicles (OMVs) are nano-sized vesicles (20–200 nm) released by a diverse range of Gram-negative bacteria and enriched in protein, polysaccharide, and lipid components, including an abundance of potent immunogens.^77^ By retaining the pathogen antigenic surface composition, OMVs elicit an innate immune response as well as prime the adaptive immune response.^78^ Since a licensed OMV vaccine against *Neisseria meningitides* has been proven safe and protective in humans,^79^ OMVs as vaccine development have received more attention recently. OMVs provide an economically-favorable vaccine platform due to their relatively inexpensive preparation and high stability. Moreover, OMVs encase a broad spectrum of immunogens, providing the theoretical advantages of simultaneously priming immunity against many antigens and thereby reducing the likelihood of antigen circumvention. In 2018 WHO plague vaccine workshop, one research team intended to utilize *Bacteroides* OMVs to deliver *Y. pestis* LcrV antigen as a new vaccine candidate. In the preliminary findings, non-human primates (NHPs) intranasally immunized with LcrV-containing OMVs generated considerable anti-LcrV IgG response in sera and anti-LcrV IgA response in salivary glands and broncho alveolar fluids (BAL).^80^

## Monoclonal antibodies as therapeutic vaccines

LcrV- or F1-specific humoral immune responses alone can be effective in protection against *Y. pestis*.^81,82^ Previous studies showed that anti-LcrV or F1 monoclonal antibodies (mAbs) can passively protect mice against plague challenge.^83–85^ Intratracheal delivery of aerosolized LcrV-specific and F1-specific monoclonal antibodies (MAbs 7.3 and F1-04-A-G1) protected mice in a model of pneumonic plague.^86^ Dimitrov’s group identified one F1-specific human mAb (m252) and two LcrV-specific human mAbs (m253, m254), and demonstrated that m252 affords better protection in mice against s.c. challenge with ∼25–40 LD_50_ of *Y. pestis* CO92 than the other two mAbs.^87^ Recently, Liu et al. identified four anti-F1 mAbs. Three of the mAbs (F5C10, F6E5, and F2H5) provided different levels of protection in mice subcutaneously challenged with 600 CFU of *Y*. *pestis* 141 strain. Among then, F2H5 provided complete protection in Balb/c mice subcutaneously challenged with *Y*. *pestis* 141 strain.^88^ Collectively, it would be possible that mAbs specific to F1 or LcrV can be utilized as a fast-acting post-exposure treatment for humans against *Y. pestis* infection.

## Efficacy and safety of plague vaccine. Where is the cut-off?

Half a century ago, the USA developed and approved a formalin-killed whole cell *Y. pestis* vaccine (USP) which was used to vaccinate military during the Vietnam War.^89,90^ This vaccine afforded effective protection against bubonic plague, but the vaccine was highly reactogenic and failed to provide long-term protection and any protection against pneumonic plague,^33,89,91,92^ thus limiting its application against weaponized pneumonic plague. The rF1-V and RYpVax are safe and have passed through Phase I and II clinical trials,^27,36^ but the results of these Phase II trials are not yet available. In 2017, the FDA granted Orphan Drug status for the rF1-V plague vaccine (https://globalbiodefense.com/2017/03/10/fda-grants-orphan-drug-designation-plague-vaccine/) that is proposed for marketing in 2020, which will provide effective prophylaxis to individuals at high risk of exposure to virulent *Y. pestis*. However, concerns of inefficacy arise due to the presence of F1-negative strains in natural reservoirs that have caused fatal disease in mice and Africa green monkey.^93,94^ The Δ*caf1 Y. pestis* CO92 was not only fully virulent to mice by bubonic and pneumonic plague challenge but also surpassed immune responses mounted from live-attenuated strains or F1 subunit vaccines.^95,96^ Andrews et al. showed that immunization of sole F1 capsular antigen provided significant protection against *Y. pestis* CO92 challenge, but failed to protect mice against *Y. pestis* C12 strain (F1^-^ strain) by s.c. infection.^97,98^ Batra et al. also showed that vaccination with recombinant F1 alone failed to protect mice against *Y. pestis* S1 strain challenge by the intraperitoneal route.^45^ Altogether, these results dampen the reliability of F1 antigen as a sole antigen vaccine, despite the existence of many studies that demonstrated the immunization with F1 antigen alone,^97,99^ transfer of anti-F1 serum,^100^ or one dose of F1 formulated in poly(lactide-co-glycolide) (PLG) microparticles^101,102^ significantly afforded protection against F1+ *Y. pestis* challenge.

In addition, the existence of *lcrV* polymorphisms in the *Y. pestis* subspecies^103^ might alter the protective efficacy of vaccines only composed of LcrV and F1, although these variations in the LcrV did not alter the lethality of these strains in mice and their natural hosts so far. In consideration of this reduced efficacy, Miller et al. investigated impact of polymorphisms in the *lcrV* gene of *Y. enterocolitica* on plague protective immunity. Their results showed that polyclonal or monoclonal antibodies raised against LcrV of *Y. pestis* KIM D27 were unable to block the type III injection of *Y. pestis* expressing LcrV(W22703) from *Y. enterocolitica* O:9 strain W22703 or LcrV(WA-314) from O:8 strain WA-314. Fortunately, the results showed these strains were unable to escape LcrV-mediated plague protective immunity in the intravenous challenge model.^104^ Thus, combination of multiple antigens was tested to prevent this risk.^68,70,105^ Studies have suggested that vaccine efficacy may be different when measuring protection against bubonic or pneumonic plague infection. Th1-skewed and Th17-skewed immune responses from vaccines provide better protection against the pulmonary *Y. pestis* infection than Th2-skewed responses from subunit vaccine.^106–111^ Therefore, vaccines formulations employing different Th1-skewing and Th17-skewing adjuvants, such as MPLA^112^ or CAF01^50,113^ could potentially achieve greater protection.

The live attenuated *Y. pestis* vaccines, EV series, made in 1920s, have been administered to millions of people in Madagascar, Indonesia, Vietnam, and the Soviet Union.^114,115^ Single dose prime vaccination with the EV NIIEG live vaccine was able to induce immune responses that lasted one year against bubonic and, to some extent, pneumonic plague.^25,116^ Theoretically, the live EV series of vaccines are much better than the killed vaccine. However, the live vaccines were somewhat pathogenic in non-human primates and reactogenicity in humans,^91,117–119^ retained virulence when administered intranasally (i.n.) and intravenously (i.v.)^107,118,120^ or to persons carrying hemochromatosis.^121^ The lack of transparent protection and safety data in previous large-scale human immunization, and the lack of genetically uniformity of the vaccine strain due to many passages,^118^ has prevented the EV series of vaccines from gaining worldwide acceptance, especially in the US and Europe.^89^ As research efforts continue to construct live attenuated *Y. pestis* vaccine strains with specifically defined mutations, so do reaching the goal of balancing safety with protective efficacy. Moreover, the rational alteration of a live attenuated *Y. pestis* vaccine strains for induction of both humoral and cell-mediated immune responses toward several *Y. pestis* antigens will theoretically provide stronger protection than vaccines based on a combination of a few antigens.

Recently, the WHO conceptualized a Plague Vaccine Target Product Profile (TPP) at the WHO Plague Vaccine Workshop in 2018.^80^ In this map, there exists at least 17 plague vaccine candidates in the pipeline, including subunit (F1/V-based with adjuvant), bacterial vector-based (e.g., OMV-delivered, *Salmonella*-expressed), viral vector-based (e.g., Ad5-based, Chad-based), *E. coli* T4 bacteriophage-based, and live attenuated (e.g., *Y. pseudotuberculosis*-based or *Y. pestis*-based) vaccines expressing one or several primary antigens of *Y. pestis* (e.g., F1 capsular protein antigen, LcrV antigen, YscF antigen, and/or pesticin coagulase), which have been tested in different animal models. Two of these candidates have completed a Phase 2 clinical trial and are moving toward FDA licensure, and several candidates have plans to enter clinical trials in 2019.

The requirements and considerations of the WHO TPP^80^ for a prophylactic plague vaccine include elicitation of long lasting immunity, and feasible administration in populations living in endemic areas or health workers involved in plague outbreak investigation or surveillance. The requirements and considerations for a therapeutic vaccine includes elicitation of a rapid protective immunity after the first dose within a narrow window, and protection of individuals in outbreak areas to block transmission chains. Mechanisms of protective immunity are complex and vary depending on the vaccine design and the route of administration, in addition to variations in the immune response induced by the intrinsic attributes of different vaccine candidates. Many recent studies have demonstrated that heterologous prime-boost immunizations could potentially be more immunogenic than homologous prime-boost immunizations.^70,122–125^ Thus, combinations of different vaccine forms using a heterologous primer-boost strategy, such as a subunit vaccine with a live attenuated *Y. pestis* vaccine or live vectored plague vaccine, might overcome current limitations of plague vaccines and would effectively prevent the potential plague outbreak.
